# Supplementary material for: Harmful and Harmless Soil-Dwelling Fungi Indicate Microhabitat Suitability for Off-Host Ixodid Ticks
Source: Microorganisms. 2024 Mar 19;12(3):609. doi: 10.3390/microorganisms12030609 (PMC10975982; doi:10.3390/microorganisms12030609)
Supplement: Supplementary file 1 [file microorganisms-12-00609-s001.zip › Supplementary Figure 1.pdf]

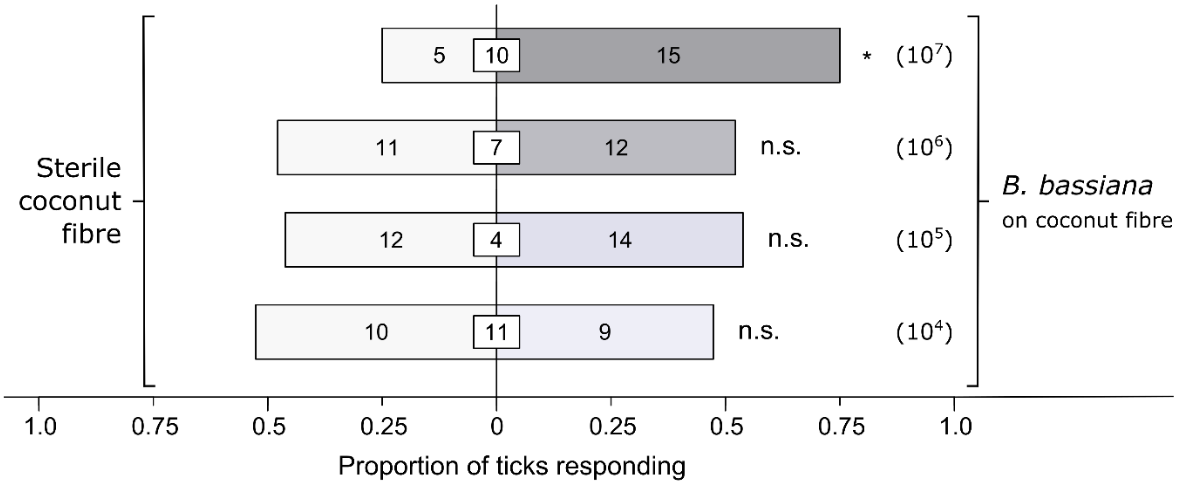

Supplementary Figure S1. Effect of dose (conidia/mL) of a *Beauveria bassiana* spore suspension on behavioral responses of female black-legged ticks, *Ixodes scapularis.* In olfactometers bioassays (Fig. 1), ticks were offered a choice between coconut fibre treated, or not (control), with a 1-mL *B. bassiana* spore suspension of 10^4, 10^5, 10^6, or 10^7 conidia/mL incubated 24 h before bioassays. The control coconut fibre was treated with sterile 0.05% Tween80 (1 mL). Numbers in bars represent the total number of ticks choosing a stimulus, and numbers in white inset boxes represent the total number of non-responding ticks. Asterisks indicate significant arrestment behavior on coconut fibre treated with the fungal spore suspension (exact binomial tests; * $p < 0.05$; n. s. = not significant).
